# Evaluation of Pain in the Pediatric Patient Admitted to Sub-Intensive Care: What Is the Evidence? A Scoping Review

**DOI:** 10.3390/epidemiologia6010009

**Published:** 2025-02-20

**Authors:** Antonio Bonacaro, Carlotta Granata, Chiara Canini, Lucrezia Anderle, Federica Ambrosi, Maria Chiara Bassi, Giacomo Biasucci, Andrea Contini, Giovanna Artioli, Elisa La Malfa, Massimo Guasconi

**Affiliations:** 1 Department of Medicine and Surgery, University of Parma, 43121 Parma, Italy; antonio.bonacaro@unipr.it (A.B.); chiara.canini@unipr.it (C.C.); giacomo.biasucci@unipr.it (G.B.); giovanna.artioli@unipr.it (G.A.); elisa.lamalfa@unipr.it (E.L.M.); 2 Azienda USL of Piacenza, 29121 Piacenza, Italy; c.granata@ausl.pc.it (C.G.); a.contini@ausl.pc.it (A.C.); 3 Azienda Provinciale per i Servizi Sanitari of Trento, 38123 Trento, Italy; lucrezia.anderle@apss.tn.it (L.A.); federica.ambrosi@apss.tn.it (F.A.); 4 Azienda USL—IRCCS of Reggio Emilia, 42122 Reggio Emilia, Italy; mariachiara.bassi@ausl.re.it

**Keywords:** pain assessment tools, pain assessment, pediatric intensive care unit, pediatric sub-intensive care unit, scoping review

## Abstract

Background and Objectives: Inadequate pain treatment in pediatric patients can cause long-term physical and psychological issues. Accurate detection of pain presence and intensity is crucial, especially in Neonatal and Pediatric Sub-Intensive Care Units. Due to uncertainties about the best pain assessment tool in these settings, it is necessary to review the literature to identify the available evidence. Methods: A scoping review was performed to address the question: What tools are available for pain assessment in non-sedated, non-intubated pediatric patients in sub-intensive care? Searches were conducted in databases including PubMed, Scopus, Embase, CINAHL, Cochrane Library, Web of Science, Open Dissertation, as well as CENTRAL and ClinicalTrials.gov registries. Results: The review included 27 studies, revealing various tools for pain assessment in pediatric sub-intensive settings. All studies favored the use of multidimensional scales, combining physiological and behavioral indicators. Conclusions: This review offers a comprehensive overview of the tools for pain assessment in pediatric patients in sub-intensive care settings but does not determine a single best tool. Most studies focused on the validation, translation, and adaptation of these tools. Further research is needed on the practical application of these tools and the perceptions of those administering them.

## 1. Introduction

Pain has been recognized since 1999 as the fifth vital parameter [1] and is defined by the International Association for the Study of Pain (IASP) as “an unpleasant sensory and emotional experience associated with, or resembling that associated with, actual or potential tissue damage” [2].

Pain is a symptom shared by patients of all ages, but in children, its assessment is particularly challenging and has for years been underestimated and inadequately treated, while in infants it has long been ignored [2,3,4]. However, in the pediatric setting, pain is a frequent symptom: about 40–80% of hospitalized critically ill children present pain due to trauma, medical procedures, invasive devices, and illness-induced discomfort [5,6,7].

Over the years, numerous studies have been conducted that have refuted previous beliefs about pain in children, highlighting how inadequate treatment of pain in the pediatric population can lead to long-term physical and psychological consequences [3,8].

The World Health Organization (WHO) has initiated global efforts to advance pediatric pain management in low- and middle-income countries, where resources and proper training to tackle this issue are often insufficient [9].

It is therefore crucial to detect the presence and intensity of pain, and it is essential to do so with appropriate instruments that take into account several factors, such as gestational age, pathology, cognitive developmental stage, behavioral, and relational factors [3,8,10].

In many countries, significant programs and studies have been launched to address this challenge. For instance, in the United States, the American Academy of Pediatrics (AAP) has published detailed guidelines on pediatric pain management, promoting the use of standardized tools. In the United Kingdom, the National Institute for Health and Care Excellence (NICE) has developed recommendations for the treatment of acute and chronic pain in children, integrating pharmacological approaches with non-pharmacological strategies, such as music therapy and psychological support. The use of standardized tools for pediatric pain assessment is also emphasized in Italy [11,12,13].

In children who are able to cooperate, the guidelines recommend the use of self-assessment scales, in which the perception of pain experienced is reported against a figurative model or numerical values, while in children who are unable to express themselves verbally, heteroassessment scales are used, where the presence of pain is detected through physiological and/or behavioral indicators [14,15].

There are several tools for assessing pain in children; in particular, the literature [3,8,14] suggests the use of the “Face, Legs, Activity, Cry, Consolability” (FLACC) scale, an observer-rated tool which assesses acute and prolonged pain from a behavioral perspective, for children less than 3 years old [16,17,18]; the use of the Wong–Baker scale, a self-report tool that uses a range of facial expressions to indicate the intensity of pain, for children between the ages of 3 and 7 years old [19,20]; and the numeric scale, Numeric Rating Scale (NRS) or Verbal Numeric Scale (VNS), self-report scales that ask the patient to grade pain from 0 (no pain) to 10 (worst pain), for children older than 8 years of age [21,22].

For children with cognitive impairment, the FLACC-R [23] and “Non-communicating Children’s Pain Checklist Revised” (NCCPC—R) [24] scales are recommended. The FLACC-R and the NCCP-R are observer-rated tools which assess pain from a behavioral perspective and differ from FLACC and NCCP because some item descriptions have been adapted to the atypical behaviors of cognitively impaired children. With regard to the Pediatric Intensive Care Unit (PICU) and Neonatal Intensive Care Unit (NICU), it is recommended to use the COMFORT–Behavior scale (COMFORT—B) [25]. This scale measures eight behavioral parameters related to pain, distress, and sedation in pediatric patients; however, given the two-minute time required for assessment, it is often used and applied incorrectly by nursing staff due to a lack of time [26]. The Behavioral Pain Scale (BPS) seems to be suitable for the PICU and NICU context and could obviate the prolonged observation time. The BPS is a behavior rating scale that evaluates three behavioral domains: facial expression, movements of upper limbs, and compliance with ventilator [27].

In the context of the Neonatal Sub-Intensive Care Unit (Sub-NICU) and Pediatric Sub-Intensive Care Unit (Sub-PICU), which receives pediatric patients who are not critical enough to be admitted to PICU and NICU, but at the same time are unsuitable for pediatric care and thus be able to use FLACC, Wong–Baker, and NRS/VRS, it is unclear which instrument is best to detect pain.

The purpose of this review is therefore to map the literature in order to identify the available evidence regarding pain detection in the sub-NICU and sub-PICU setting.

## 2. Materials and Methods

### 2.1. Design

This article presents the results of a scoping review [28,29,30,31] conducted using the JBI guidelines in agreement with the protocol published by Granata et al. [32] and registered on Open Science Framework (https://doi.org/10.17605/OSF.IO/8KBRQ).

The scoping review design was chosen because the literature regarding the topic of interest seems to be complex and heterogenous [29,30]. The results are reported in accordance with the PRISMA extension for scoping review guidelines (PRISMA-ScR) [33,34].

### 2.2. Review Methods

#### 2.2.1. Review Question

The research question was formulated according to the PCC mnemonic, and it is as follows: “What tools are available for pain assessment (Concept) in pediatric patients not sedated and not intubated (Population) admitted to sub-intensive care (Context)?

#### 2.2.2. Inclusion Criteria

Participants

The population included in the study sample is the pediatric population starting with infants, including preterm infants, up to 18 years of age. The 18-year limit was chosen because it is the one identified by the Italian health regulation and it is indicated as a pediatric age limit by several WHO documents as well [9,35]. However, studies were not excluded if they included patients older than 18 years if local health regulations allow pediatric hospitalization for patients older than 18 years. Studies involving non-sedated and non-intubated patients were included.

Concept

Articles concerning pain detection that examined assessment instruments but also the perceptions and opinions of practitioners using them were included.

Context

Studies that explored sub-PICU and sub-NICU settings were initially considered, while out-of-hospital, PICU/NICU, and emergency department settings were excluded. While conducting the review, it became necessary to modify the above criteria [31]. In fact, it was realized that sub-PICU and sub-NICU are often equated with PICU and NICU, so articles conducted in PICU and NICU that concerned non-sedated and non-intubated children with pathological features of sub-ICU were also included in the review.

Types of sources

This scoping review considered quantitative studies, qualitative studies, and mixed-methods studies. In addition, systematic reviews that meet the inclusion criteria were considered. Moreover, gray literature, indications and guidelines of scientific societies, reports, and conference abstracts were considered for inclusion in the review.

#### 2.2.3. Search Strategy

The source search was completed on 15 December 2022.

The search strategy was formulated with the support of a documentalist and was revised after doing a preliminary search in PubMed [29,30] The final strategy for PubMed is depicted in Appendix A.

The search was conducted using the online databases PubMed, Scopus, Embase, CINAHL, Cochrane Library, Web of Science, Open Dissertation (EBSCO), and DOAJ. CENTRAL e ClinicalTrials.gov registries were also included.

A citation search starting from the references of the included articles and a grey literature search were performed.

#### 2.2.4. Study/Source of Evidence Selection

The results obtained from the databases were entered into Rayyan [36], software that supports researchers in identifying duplicate records and screening.

The phase of study selections undertaken can be divided into three steps: (i) The title and abstract of the records remaining after duplicate records were eliminated were evaluated based on the inclusion criteria. (ii) Full-text articles obtained from phase one were analyzed in order to determine their actual relevance or not to the research question. Full-text studies that did not meet the inclusion criteria were excluded. (iii) The flow of included and excluded records was represented in a PRISMA flow diagram [33,34].

All stages of selection were conducted blindly by two groups of independent researchers; any disagreements that arose between the groups of researchers were resolved through discussion and the support of a third reviewer.

Reference lists of included articles were then analyzed and a snowball search was performed in order to identify any additional resources.

Finally, a search of the websites of major pediatric scientific societies was performed.

#### 2.2.5. Data Extraction

The characteristics of the included studies have been included in a table [29,30,31] constructed a priori and redefined based on the results obtained.

Article title, authors, journal, year of publication, study origin, study design adopted, number of participants, assessment tools used, study population, study concept, and study context were entered in the table.

#### 2.2.6. Data Analysis and Presentation

Researchers performed data extraction and analysis from the study results in relation to the research question and the Population–Context–Concept (PCC) [29,30,37].

Data inherent in the studies were described and represented graphically in relation to type of study included, temporal distribution of studies included, origin of studies, population involved in the studies, pain assessment tools cited in the literature, and the context of studies.

## 3. Results

**Figure 1:**
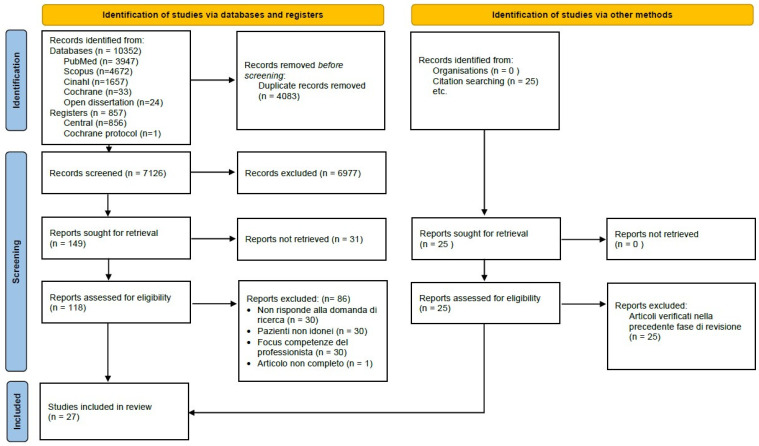
PRISMA flow diagram [33].

From the database search, 11,209 articles were found, but as shown in the PRISMA diagram (Figure 1), 4083 articles were eliminated because they were duplicates.

Of the remaining 7126 records, after reading the title and abstract, only 149 studies were considered potentially relevant to the research question. Subsequently, the full text of 118 articles was analyzed independently by the two groups of researchers in order to decree their final adherence to the object of investigation. For 31 studies, however, it was not possible in any way to find the article and thus proceed to read the full text.

Finally, 27 articles were included in the review because they answered the research question and met the inclusion and exclusion criteria (Appendix B).

### 3.1. Characteristics of Included Studies

#### 3.1.1. Study Design

**Figure 2:**
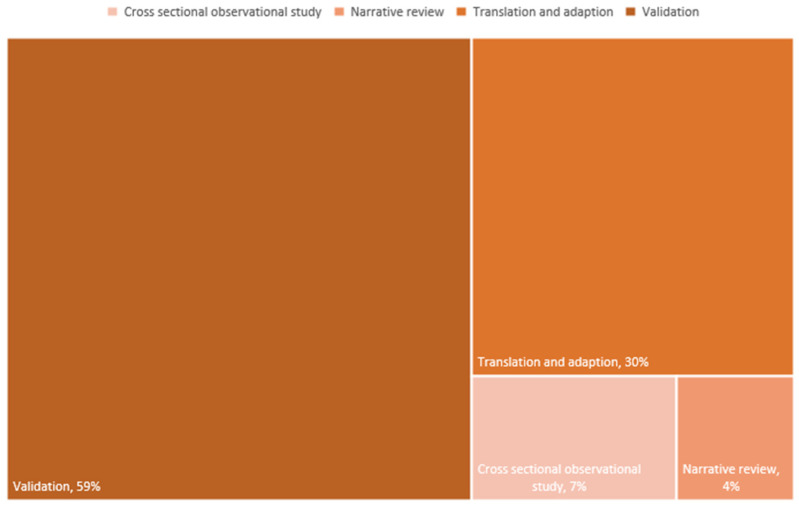
Tree diagram of study designs with their percentages.

As depicted in Figure 2, most of the included articles (n = 16, 59%) were studies aimed at validating tools [38,39,40,41,42,43,44,45,46,47,48,49,50,51,52,53]. Eight (30%) articles, on the other hand, concerned studies of the translation and adaptation of existing instruments, i.e., translation and validation of scales in new cultural contexts [54,55,56,57,58,59,60,61]. Finally, two (7%) articles were cross-sectional observational studies [62,63] and one (4%) was a narrative review [64].

#### 3.1.2. Time Distribution of Studies

**Figure 3:**
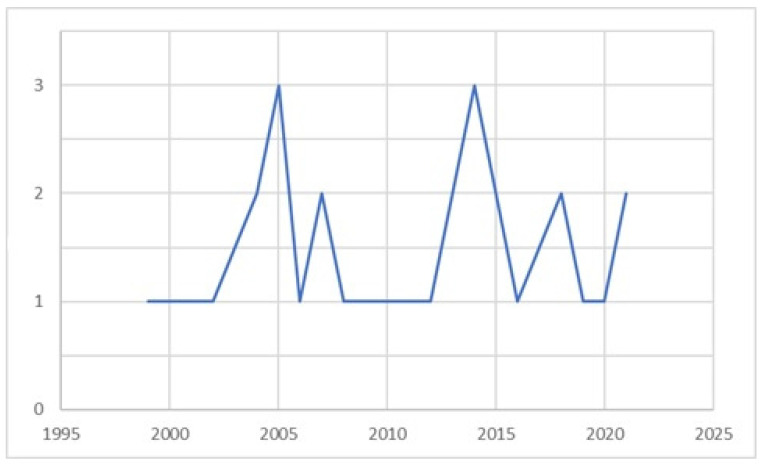
Time distribution of included studies.

The included articles were published in a time range from 1999 to 2021 (Figure 3).

In particular, one (4%) study was from 1999 [38], eight (30%) studies were published between 2000 and 2005 [40,41,45,48,50,57,62,64], six (22%) between 2006 and 2010 [39,43,44,47,51,61], five (18%) between 2011 and 2015 [42,46,49,55,59], five (18%) were published between 2016 and 2020 [52,54,58,60,63], and two (7%) in 2021 [53,56].

#### 3.1.3. Provenance of Included Studies

**Figure 4:**
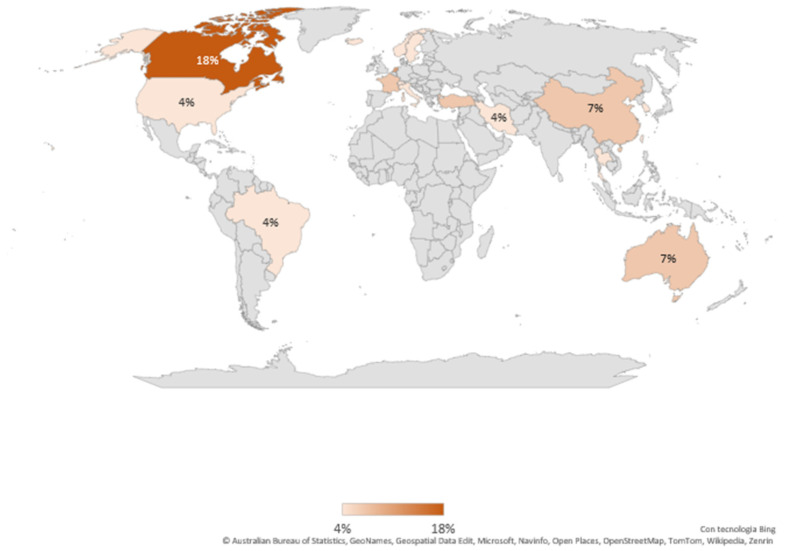
Geographic distribution of included studies with their percentages.

Most of the studies (n = 10, 37%) were conducted in Europe [39,40,41,47,50,51,57,58,61,63], followed by Asia (n = 8, 30%) [46,52,53,54,56,59,60,62] and from North America (n = 6, 22%) [38,42,43,44,45,49]. Two (7%) articles were from Oceania [48,64] and one (4%) from Latin America [55] (Figure 4).

#### 3.1.4. Population

All included articles have pediatric patients with clinical features attributable to sub-intensive care as their reference population.

With regard to age, most of the included studies considered neonates. Specifically, seventeen (63%) articles included both term and preterm infants [38,40,42,45,46,48,49,51,54,56,57,58,59,60,61,62,63], eight (30%) studies included preterm infants [39,41,43,44,47,52,53,55], one (4%) study included both term and preterm infants and children up to 3 years old [50], and the review of Ramelet et al. takes into consideration pediatric patients of all ages [64].

#### 3.1.5. Context of the Studies

Much of the studies are conducted within NICUs, while the Sub-ICU context appears to be rarely mentioned.

It appears from the literature that this context tends to be unified with the ICU, as the difference between the two operating units is difficult to delineate in terms of admission criteria.

Only in of reviewed two studies was there a clear distinction made between the Sub-PICU and the PICU [45,55], In the other studies, the focus is more on the characteristics and criticality of the patient, as opposed to the patient’s context.

#### 3.1.6. Scales

Multiple instruments used for the assessment of children’s pain in the ICU setting emerge from the literature, some of which are cited more. In Table 1, the multidimensional scales found in the literature are presented and in Table 2 the single-dimensional scales are presented.

An element in agreement with all the studies analyzed is the preference for the use of multidimensional scales involving the association of both physiological and behavioral indicators.

### 3.2. Premature Infant Pain Profile (PIPP)

One of the scales analyzed is the Premature Infant Pain Profile (PIPP). This is an instrument that assesses procedural and postoperative pain in the preterm and term infant by taking into consideration both physiological and behavioral indices. It has been validated, translated, and analyzed within cross-sectional studies. In multiple validation studies, its good validity and reliability have been emphasized [38,61]. Another result that goes to confirm the validity and reliability of the PIPP emerged from the validation process that focused on comparing instruments that assess behavioral and physiological parameters with instruments focused only on the behavioral aspect. Specifically, the PIPP and the Neonatal Infant Pain Scale (NIPS), multidimensional scales, were compared with the Neonatal Facial Coding System (NFCS) and the Doleur Aigue du Nouveau-né scales (DAN), which assess only behavioral factors. This study, moreover, showed that instruments that analyze physiological and behavioral indicators are better than those scales that consider only the behavioral parameter [53]. Similar analysis was conducted in the Korean cross-sectional study by Ahn et al. [62]. In this case, comparison was made with the multidimensional scale Crying, Requires Oxygen, Increased vital signs, Expression, Sleepless (CRIES) that assesses postoperative pain in the preterm infant (32–36 weeks) and with the FLACC scale that assesses acute and prolonged pain in children younger than 3 years of age by observation of behavioral aspects alone. CRIES and FLACC measure pain reliably and have been found to be clinically appropriate. In contrast, further studies are recommended for PIPP on the time aspect of data collection and clinical feasibility [62]. Comparison with “pain monitor” surveys conducted within another cross-sectional study showed that the PIPP scale tends to underestimate procedural pain [63].

### 3.3. Premature Infant Pain Profile-R (PIPP-R)

Another scale that emerged from the analyzed studies is the Premature Infant Pain Profile-R (PIPP-R). This is a revision of the PIPP scale in which modifications have been made with the aim of simplifying the calculation of the final score that takes into consideration the behavioral aspect along with the physiological aspect. It is considered a reliable and valid scale [42,58,60]. Compared with the PIPP scale, it appears to be more user-friendly [49].

### 3.4. COMFORT Scale and COMFORT-Neo

The COMFORT scale assesses pain both behaviorally and physiologically in intensive settings in children aged 0–18 years. It has been regarded as useful and reliable [39,51]. The quality of this scale is also reaffirmed in the narrative review by Ramelet et al., who analyze it by comparing it with multiple instruments in the literature [64].

An evolution of the COMFORT scale is the “COMFORT-neo” scale which, when compared with the NRS scale in the validation study by Van Dijk et al., turns out to have preliminary reliability [51].

### 3.5. Other Scales Who Consider Both Behavioral and Physiological Aspects

Other scales that take into consideration both behavioral and physiological aspects that were subject to validation or translation and found to have positive reliability were the Bernese Pain Scale for Neonates (BPSN) which assesses acute pain in preterm and term infants, the Pain Assessment Tool (PAT) scale which assesses postoperative pain in term and preterm infants, the Faceless Acute Neonatal Pain Scale (FANS) which was compared with the Doleur Aigue du Nouveau-né (DAN) scale, the Pain Assessment in Neonates (PAIN) scale that assesses procedural pain and was compared with the Neonatal Infant Pain Scale (NIPS) and CRIES scales, the multidimensional PASPI scale, the COVER scale that assesses pain in preterm and term infants, and the Clinical Pain Scale for Preterm Neonates (CPSPN) scale that was compared with the PIPP-R scale [40,45,46,47,48,52,56].

### 3.6. Scales That Consider Only the Behavioral Aspects

Two scales that consider only the behavioral aspects were found. They are the Echelle Doleur Inconfort Noveau-né (EDIN) scale, which appears to be an appropriate instrument restricted, however, to critical preterm infants only [41,55]. The other behavioral scale is the Behavioral Indicators of Infant Pain scale (BIIP) that assesses procedural pain in preterm infants later translated into Chinese (C-BIIP), also considered as reliable [43,44,54].

### 3.7. Scales Descripted by Ramelet et al. in Their Narrative Review [64]

Within the narrative review by Ramelet et al. [64], 28 pain detection scales are analyzed: 11 confined to the neonatal population, 11 for children aged 0–3 years, and six for children older than 12 months. Some of these scales have already been covered in the previous studies described; some assess both behavioral and physiological aspects such as the PIPP, PAIN, PAT, CRIES, NIPS, and COMFORT scales; others such as the EDIN, DAN, NFCS, and FLACC scale take into consideration only the behavioral aspect [64]. The other multidimensional scales that assess aspects of behavior and physiology are the Scale for Use in Neonates (SUN) which assesses acute pain in premature infants, the Distress Scale for Ventilated Infants (DISVNI) which assesses procedural pain, the Neonatal-Pain, Agitation and Sedation Scale (N-PASS) used in infants even during sedation, the Modified Infant Pain Scale (MIPS) used in children aged 0 to 2 years for the assessment of post-surgery pain, the Preverbal, Early Verbal Pediatric Pain Scale (PEPPS), used for children aged one to two years, the Children’s Hospital of Eastern Ontario Pain Scale (CHEOPS) which assesses pain in children aged one to five years undergoing minor surgery, the Nursing Assessment of Pain Intensity (NAPI) scale used in children up to 3 years of age in post-surgery, and the Objective Pain Scale (OPS) used for the assessment of postoperative pain most in children older than 3 years. The other scales that assess only physiological parameters are the Liverpool Infant Distress Scale (LIDS) which assesses postoperative pain in infants, the Infant Body Coding System (IBCS) which assesses pain in newborns, the Pain Observation Scale for Young Children (POCIS) which assesses postoperative pain in children aged one to four years, the Toddler-Preschooler Postoperative Pain Scale (TPPS) used to assess postoperative pain in children aged one to five years, the Child Facial Coding System (CFCS) which assesses postoperative pain in children aged one to six years, the Postoperative Pain Score (POPS) scale which assesses postoperative pain by associating neurological and behavioral aspects, the CHIPPS scale which assesses postoperative pain in children aged up to five years, the Behavioral Pain Score (BPS) scale which assesses pain during sedation in children up to three years, and the Modified Behavioral Pain Score (MBPS) scale which assesses pain in children aged four to six months [64].

## 4. Discussion

### 4.1. Summary of Evidence

Assuming that pain has been recognized since 1999 as the fifth vital parameter [1] and that it is an essential component of health care [65], from the literature review, it is possible to see the presence of numerous scales that can be used in the detection of pediatric patient pain within the intensive care setting. Some of them take into account only the behavioral aspect; others go to link the latter with the physiological aspect thus associating parameters such as SpO_2_ or HR, for example.

The use of multidimensional scales is recommended because the association of physiological and behavioral responses makes the response more comprehensive considering the difficulty in detecting pain, especially in the preverbal pediatric population [66].

It is difficult to say which instrument is the best as from the analysis of the literature it is possible to see that most of the included studies are processes of validation, translation, and adaptation of instruments.

The search for new instruments, therefore, shows how interest is increasing on the topic of pain in pediatric patients in the Sub-PICU and PICU clinical areas and how the search for an instrument that can comprehensively assess and detect pain and ensure homogeneity is needed.

At the same time, however, this result highlights the lack of studies in the literature regarding the application of the instrument or studies aimed at analyzing practitioner thinking regarding the usability of the instrument itself.

The progressive interest in the topic can be seen by visualizing the temporal distribution of the included studies. In 1999, there was only one study inherent to this topic, while since the 2000s, it has been possible to detect the presence of more studies: eight between 2000 and 2005, five between 2011 and 2015, five between 2016 and 2020, and two in 2021.

During the period we analyzed, a few studies were recorded during the COVID-19 pandemic phase [67]. In these cases, it must be remembered that the emergency network underwent major changes [67] as well as the epidemiology of the diseases managed and the patients admitted to the emergency department. In the post-pandemic phase, major changes were recorded [68] and this may have altered the usefulness of the scales during pandemics, which is why it is necessary to continue this type of study in subsequent years.

The increased sensitivity to the topic of pain in pediatric patients in general could be related to the fact that until a few years ago, pain in children was underestimated and inadequately treated, as infants and children were thought to experience less pain than adults, and the literature on the subject was rather sparse [2,3,4]. In recent years, multiple studies have been conducted that have led to a reconsideration of knowledge about the perception of neonatal pain, showing how previous beliefs were wrong and how pain can bring physical and psychological consequences if not properly treated [3,19].

Geographically, it was found that most of the studies are mainly from developed or developing countries; this analysis shows that in much of the world, the subject of this scoping review is not addressed at all, so it would be interesting to analyze this in other contexts as well.

Regarding the population, the focus is mainly on preterm and term infants, thus on a segment of the preverbal population that is in the condition of not being able to express their experience of pain in words, while only two studies also involve infants.

Regarding the setting, as already highlighted in the results, it was difficult to clearly delineate the division between intensive and sub-intensive care as they are often interrelated. Another aspect that emerges is that many scales go to assess pain [3,8].

### 4.2. Limitations

This study has some limitations. Although all included studies were on patients who met the inclusion criteria, few articles regarding sub-intensive care were found. In addition, 31 full texts could not be found, so interesting data may have been missed.

## 5. Conclusions

This scoping review provides a broad overview of tools that can be used to assess pain in pediatric patients in intensive and sub-intensive care settings but does not allow us to determine which one is best. Therefore, it would be appropriate to explore this topic further with systematic reviews following the “COSMIN” Guidelines.

Since there are more analyses conducted on validation, translation, and adaptation studies, it would be necessary to carry out further research on the application of the instrument itself or on the perception by those administering the scale that involve the pediatric patient more, not only in the neonatal period, by specifying more precisely the setting of origin.

The identification and proper management of pain in neonates and children in sub-intensive care settings contribute to improving the well-being and quality of life of young patients, reducing the risk of long-term physical and psychological consequences. For healthcare providers, this study offers insights into the adoption of more appropriate assessment tools for specific settings, enhancing clinical approaches and promoting personalized care. Moreover, the standardization of assessment tools and ongoing training can help reduce disparities in pain management across countries and healthcare facilities, increasing the efficiency and equity of the healthcare system. On a systemic level, the integration of evidence-based practices can strengthen global healthcare infrastructure, improving health outcomes and reducing long-term costs by preventing complications and improving the quality of care.

For future research, it is hoped that from the geographical point of view there may be greater extension by preferring the application of multidimensional instruments that analyze the physiological aspect associated with the behavioral aspect.

## Figures and Tables

**Table 1:** Multidimensional scales description.

Multidimensional Scales	Physiological Indicators	Behavioral Indicators	Purpose	Populations
PIPP: Premature Infant Pain Profile	HR, SaO_2_	Facial expressions	Procedural and postoperative pain	Preterm and term infant
NIPS: Neonatal Infant Pain Scale	Breathing pattern State of arousal	Facial expressions Cry/verbal expressions Body movements	Procedural pain	Neonates
CRIES: Crying, Requires Oxygen, Increased vital signs, Expression, Sleepless	HR, BP, SaO_2_	Facial expressions Cry/verbal expressions	Postoperative pain	Preterm infant (32–36 weeks)
PIPP-R: Premature Infant Pain Profile-R	A revision of the PIPP scale in which modifications have been made with the aim of simplifying the calculation of the final score.			
COMFORT	BP, HR	Facial expressions Cry/verbal expressions Body movements	Discomfort caused by pain	Children aged 0–18 years
COMFORT-neo	Evolution of the COMFORT scale used specifically in the neonatal intensive care unit setting			
BPSN: Bernese Pain Scale for Neonates	RR, HR, SaO_2_	Facial expressions Cry/verbal expressions Body movements	Acute pain	Preterm and term infants
PAT: Pain Assessment Tool	HR, SaO_2_, BP	Facial expressions Cry/verbal expressions Posture	Postoperative pain	Term and preterm infants
FANS: Faceless Acute Neonatal Pain Scale	HR, SaO_2_	Cry Body movements	Acute pain	Preterm infants
PAIN: Pain Assessment in Neonates	HR, SaO_2_	Facial expressions Cry/verbal expressions Body movements	Procedural pain	Neonates
PASPI	HR	Facial expressions Body movements Sleep–wake state transition	Pain	Preterm infants
COVERS	HR, BP, O_2_ requirement	Expression, resting, and signaling distress	Procedural pain	Term and preterm infants
CPSPN: Clinical Pain Scale for Preterm Neonates	BP, HR, SaO_2_, Breathing pattern	Facial expressions Cry/verbal expressions Body movements	Procedural pain	Preterm infants
SUN: Scale for Use in Neonates	HR, SaO_2_, Breathing pattern	Facial expressions Body movements	Acute pain	Preterm infants
DISVNI: Distress Scale for Ventilated Infants	HR, BP, SaO_2_	Facial expressions Body movements	Acute and procedural pain	Neonates
N-PASS: Neonatal-Pain, Agitation and Sedation Scale	HR, RR, BP, SaO_2_	Facial expressions Cry/verbal expressions Body movements	Acute pain	Neonates
MIPS: Modified Infant Pain Scale	HR, BP, SaO_2_	Facial expressions Cry/verbal expressions Body movements	Postoperative pain	Term and preterm infants
PEPPS: Early Verbal Pediatric Pain Scale	HR	Facial expressions Cry/verbal expressions Body movements	Pain	Children between the ages of one and two
CHEOPS: Children’s Hospital of Eastern Ontario Pain Scale	HR, BP, Breathing pattern	Facial expressions Cry/verbal expressions Body movements	Pain	Children ranging in age from 1 to 5 years old
NAPI: Nursing Assessment of Pain Intensity	HR, SaO_2_, Breathing pattern	Facial expressions Cry/verbal expressions Body movements	Postoperative pain	Children up to three years of age
OPS: Objective Pain Scale	BP	Cry/verbal expressions Body movements	Postoperative pain	Children over the age of three

**Table 2:** Single-Dimensional scales description.

Single-Dimensional Scale	Behavioral Factors	Purpose	Populations
NFCS: Neonatal Facial Coding System	Facial expressions	Procedural pain	Child between the ages of 0 and 18 months
DAN: Doleur Aigue du Nouveau-né scales	Facial expressions Cry/verbal expressions Body movements	Acute pain	Term and preterm infants
FLACC	Facial expressions Cry/verbal expressions Body movements	Acute and prolonged pain	Children younger than 3 years of age
EDIN: Echelle Doleur Inconfort Noveau-né	Facial expressions Cry/verbal expressions	Prolonged pain	Preterm infants between 25 and 36 weeks gestational age
BIIP: Behavioral Indicators of Infant Pain scale	Sleep–wake rhythm Body movements	Acute and procedural pain	Preterm infants
C-BIIP	BIIP version of the scale in its Chinese version		
LIDS: Liverpool Infant Distress Scale	Facial expressions Cry/verbal expressions Body movements	Postoperative pain	Neonates
IBCS: Infant Body Coding System	Body movements	Pain	Neonates
POCIS: the Pain Observation Scale for Young Children	Body movements Facial expressions	Postoperative pain	Children from one to four years old
TPPS: Toddler-Preschooler Postoperative Pain Scale	Facial expressions Cry/verbal expressions Rub or touch painful area	Postoperative pain	Children who are between the ages of 1 and 5 years old
CFCS: Child Facial Coding System	Facial expressions	Postoperative pain	Children aged 1 year up to 6 years old
POPS: Postoperative Pain Score	Facial expressions Cry/verbal expressions Body movements	Postoperative pain	Children aged 1 year up to 6 years old
CHIPPS	Facial expressions Cry/verbal expressions Body movements	Postoperative pain	From the newborn to the child with 5 years of age
BPS: Behavioral Pain Score	Facial expressions Cry/verbal expressions Body movements	Patients undergoing invasive and noninvasive mechanical ventilation	All ages
MBPS: Modified Behavioral Pain Score	Facial expressions Cry/verbal expressions Body movements	Procedural pain	Infants 4 to 6 months old

## Data Availability

Data sharing is not applicable to this article.

## Appendix A

PubMed search strategy

PubMed	(“Infant, Newborn”[Mesh] OR “infant”[Mesh] OR “Child”[Mesh] OR “child” OR “children” OR “newborn” OR “newborns” OR “baby” OR “babies” OR “pediatric” OR “paediatric” OR “pediatrics” OR “paediatric” OR “infant” OR “infants” OR “neonate” OR “neonates”) AND (“Pain”[Mesh] OR “Pain Measurement”[Mesh] OR “pain” OR “pains” OR “physical suffering” OR “physical sufferings” OR “ache” OR “aches” OR “pain measurement” OR “pain measurements” OR “nociception tests” OR “nociception test” OR “analgesia test” OR “analgesia tests” OR “pain assessment” OR “pain assessments” OR “pain scale” OR “pain scales” OR “pain rating scale” OR “pain intensities” OR “pain severities” OR “pain severity” OR “pain tool” OR “pain tools” OR “pain evaluation” OR “pain assessment score” OR “pain rating score”) AND (“Intensive Care Units”[Mesh] OR “Intensive Care Units, Neonatal”[Mesh] OR “Intensive Care Units, Pediatric”[Mesh] OR “Intensive Care Units” OR “Intensive Care Unit” OR “Neonatal intensive care unit” OR “ Neonatal intensive care” OR “Pediatric intensive care unit” OR “Pediatric intensive care” OR “Paediatric intensive care unit” OR “Paediatric intensive care” OR “PICU” OR “NICU” OR “high dependency unit”)

## Appendix B

Characteristics of included studies.

**Title**	**Authors**	**Journal**	**Year**	**Country**	**Design**	**Number of Participants**	**Scales**	**Population**	**Concept**	**Context**
Validation of the premature infant pain profile in the clinical setting [38]	Ballantyne, M., Stevens, B., McAllister, M., Dionne, K. and Jack, A.	The Clinical journal of pain	1999	Canada	Validation	43	PIPP	Preterm and term infants	Construct validity and reliability of PIPP	NICU
The reliability and validity of the COMFORT scale as a postoperative pain instrument in 0 to 3-year-old infants [50]	Van Dijk, M., De Boer, J. B., Koot, H. M., Tibboel, D., Passchier, J., and Duivenvoorden, H. J.	Pain	2000	Netherlands	Validation	158	COMFORT	Preterm and term infants, bambini	Validity and reliability of the COMFORT scale	Pediatric surgical intensive care unit (PSICU)
Development and initial validation of the EDIN scale, a new tool for assessing prolonged pain in preterm infants [41]	Debillon, T., Zupan, V., Ravault, N., Magny, J.-F., and Dehan, M.	Archives of disease in childhood. Fetal and neonatal edition	2001	France	Validation	76	EDIN	Preterm infants	To evaluate whether the EDIN scale can assess prolonged pain in preterm infants	NICU - Neonatal Care
Validation of the Pain Assessment in Neonates (PAIN) scale with the Neonatal Infant Pain Scale (NIPS) [45]	Hudson-Barr, D., Capper-Michel, B., Lambert, S., Mizell Palermo, T., Morbeto, K., and Lombardo, S.	Neonatal network	2002	USA	Validation	196	PAIN (Test) NIPS and CRIES (Control)	Preterm and term infants	Validity of PAIN scale	NICU and sub-intensive
Pain assessment in the neonate using the Bernese Pain Scale for Neonates [40]	Cignacco E and Mueller R and Hamers JP and Gessler P	Early human development	2004	Switzerland	Validation	12	BPSN	Preterm and term infants	Validity of BPSN scale in both ventilated and not ventilated infants	NICU
The challenges of pain measurement in critically ill young children: a comprehensive review [64]	Ramelet, A.-S., Abu-Saad, H. H., Rees, N., and McDonald, S	Australian critical care:	2004	Australia	Narrative review		PIPP, PAIN, SUN, PAT, DSVNI, N—PASS, CRIES, NIPS, EDIN, DAN, LIDS, IBCS, MIPS, POCIS, PEPPS, TPPPS, CFCS, CHEOPS, NFCS, COMFORTRIPS, POPS, NAPI, CHIPPS, BPS, MBPS, FLACC, OPS	Infants, children between 0 and 3 years old, children > 12 years old	Problems in measuring pain in critically ill children, with list of specific measures for children 0 to 3 years old	PICU
Pain assessment using CRIES, FLACC and PIPP in high-risk infants [62]	Ahn, Y., Kang, H., and Shin, E.	Journal of Korean Academy of Nursing	2005	Korea	Cross-sectional	68	CRIES, FLACC, PIPP	Preterm and term infants	Comparison among CRIES—FLACC— PIPP scales	NICU
A reliable pain assessment tool for clinical assessment in the neonatal intensive care unit [48]	Spence, K., Gillies, D., Harrison, D., Johnston, L., and Nagy, S.	Journal of obstetric, gynecologic, and neonatal nursing	2005	Australia	Validation	144	PAT (Test), CRIES (Control)	Preterm and term infants	Assessing the reliability of the PAT scale	NICU
The sensitivity of the premature infant pain profile—PIPP to measure pain in hospitalized neonates [57]	Jonsdottir, R. B. and Kristjansdottir, G.	Journal of evaluation in clinical practice	2005	Iceland	Translation and adaptation	24	PIPP	Preterm and term infants	Validation of PIPP scale in Iceland	NICU
Psychometric testing of a Norwegian version of the Premature Infant Pain Profile: an acute pain assessment tool. A clinical validation study [61]	Vederhus, B. J., Eide, G. E., and Natvig, G. K.	International Journal of Nursing Practice	2006	Norway	Translation and adaptation	123	PIPP	Preterm and term infants	Validation of PIPP scale in Norway	NICU and Neonatal care
Measurement of pain in premature infants with a gestational age between 28 to 37 weeks: validation of the adapted COMFORT scale [39]	Caljouw, M. A. A., Kloos, M. A. C., Olivier, M. Y., Heemskerk, I. W., Pison, W. C. R., Stigter, G. D., and Verhoef, A.-M. J. H.	Journal of Neonatal Nursing	2007	Netherlands	Validation	57	Adapted COMFORT scale (Test), VAS (Control)	Preterm infants	Reliability of COMFORT scale	NICU
Initial validation of the Behavioral Indicators of Infant Pain (BIIP) [44]	Holsti, L. and Grunau, R. E.	Pain	2007	Canada	Validation	92	BIIP	Preterm infants	Improve NIPS and PIPP by combining them into a single scale	NICU
Is it painful or not? Discriminant validity of the Behavioral Indicators of Infant Pain (BIIP) scale [43]	Holsti, L., Grunau, R. E., Oberlander, T. F., and Osiovich, H.	The Clinical journal of pain	2008	Canada	Validation	69	BIIP	Preterm infants	To determine whether BIIP is useful for the assessment of acute procedural pain	NICU
Taking up the challenge of measuring prolonged pain in (premature) neonates: the COMFORTneo scale seems promising [51]	Van Dijk, M., Roofthooft, D. W. E., Anand, K. J. S., Guldemond, F., De Graaf, J., Simons, S., De Jager, Y., Van Goudoever, J. B., and Tibboel, D.	The Clinical journal of pain	2009	Netherlands	Validation	286	COMFORT neo (Test), NRS (Control)	Preterm infants	Validity of COMFORT —Neo scale for prolonged pain	NICU
Validation of a neonatal pain scale adapted to the new practices in caring for preterm newborns [47]	Milesi, C., Cambonie, G., Jacquot, A., Barbotte, E., Mesnage, R., Masson, F., Pidoux, O., Ferragu, F., Thevenot, P., Mariette, J.-B., and Picaud, J.-C.	Archives of disease in childhood. Fetal and neonatal edition	2010	France	Validation	53	FANS (Test), DAN (Control)	Preterm infants	Validity of FANS scale	NICU
Validity and reliability of neonatal infant pain scale in neonatal intensive care units in Iran [59]	Sarhangi, F., Mollahadi, M., Ebadi, A., Matinzadeh, Z. K., and Tadrisi, S. D.	Pakistan Journal of Medical Sciences	2011	Iran	Translation and adaptation	68	NIPS (Test), VAS (Control)	Preterm infants	Validity and reliability of NIPS	NICU
Psychometric analysis of a Taiwan-version pain assessment scale for preterm infants [46]	Liaw, J., Yang, L., Chou, H., Yin, T., Chao, S., and Lee, T.	Journal of Clinical Nursing	2012	Taiwan	Validation	60	PASPI (Test), PIPP e VAS (Control)	Preterm and term infants	Validity of PASPI	NICU
Avaliação da dor prolongada no recém-nascido: adaptação da escala EDIN para a cultura brasileira [55]	de Souza Barbosa Dias, F., and Martins Marba, S. T.	Texto and Contexto Enfermagem	2014	Brazil	Translation and adaptation	76	EDIN	Preterm infants	Validation of EDIN in Brazil	NICU and sub-intensive
The premature infant pain profile-revised (PIPP-R): initial validation and feasibility [49]	Stevens, B. J., Gibbins, S., Yamada, J., Dionne, K., Lee, G., Johnston, C., and Taddio, A.	The Clinical journal of pain	2014	Canada	Validation	52	PIPP-R (Test), PIPP (Control)	Preterm and term infants	Validity of PIPP—R scale	NICU
Validation of the Premature Infant Pain Profile-Revised (PIPP-R) [42]	Gibbins, S., Stevens, B. J., Yamada, J., Dionne, K., Campbell-Yeo, M., Lee, G., Caddell, K., Johnston, C., and Taddio, A.	Early human development	2014	Canada	Validation	202	PIPP-R	Preterm and term infants	Validity of PIPP-R scale	NICU
La scala algometrica PIPP: valutazione dell’accuratezza per la rilevazione del dolore procedurale nel neonate [63]	Tasca, T., Spata, M., Badon, P., Pellegatta, F., Buchini, S., Monterosso, A., and Canesi, M.	Pain Nursing Magazine	2016	Italy	Cross-sectional	60	PIPP	Preterm and term infants	To evaluate the reliability of PIPP in comparison with the findings from the “pain monitor”.	NICU
Development of a Clinical Pain Scale for Preterm Neonates [52]	Woragidpoonpol, P., Tiansawad, S., Mesukko, J., and Klunklin, P.	Pacific Rim International Journal of Nursing Research	2018	Thailand	Validation	8 (phase 1), 19 (phase 2)	CPSPN (Test), PIPP-R (Control)	Preterm infants	Development and validation of CPSPN	NICU
Cultural adaptation and harmonization of four Nordic translations of the revised Premature Infant Pain Profile (PIPP-R) [58]	Olsson, E., Anderzén-Carlsson, A., Atladóttir, S. M., Axelin, A., Campbell-Yeo, M., Eriksson, M., Kristjánsdóttir, G., Peltonen, E., Stevens, B., Vederhus, B., and Andersen, R. D.	BMC pediatrics	2018	Sweden	Translation and adaptation	(video)	PIPP—R	Preterm and term infants	Validating PIPP—R in Finnish, Norwegian, Icelandic, Swedish	NICU
Psychometric Testing of the Turkish Version of the Premature Infant Pain Profile Revised-PIPP-R [60]	Taplak, A. Ş. and Bayat, M.	Journal of Pediatric Nursing	2019	Turkey	Translation and adaptation	200	PIPP-R	Preterm and term infants	Validation of PIPP-R in Turkey	NICU
Evaluation of the Reliability and Validity of the Behavioral Indicators of Infant Pain Scale in Chinese Neonates [54]	Chen, Y., Tong, Y., Xue, Z., Cheng, Y., and Li, X.	Pain management nursing:	2020	China	Translation and adaptation	369	C-BIIP (Test), FLACC (Control)	Preterm and term infants	Validation of C-BIIP in China	NICU
Assessment of four pain scales for evaluating procedural pain in premature infants undergoing heel blood collection [53]	Xie, W., Wang, X., Huang, R., Chen, Y., and Guo, X.	Pediatric research	2021	Cina	Validation	111	NFCS, DAN, NIPS, PIPP	Preterm infants	Validation of NFCS, DAN, NIPS, PIPP scales for procedural pain	NICU
Turkish validity and reliability of the COVERS pain scale [56]	İncekar, M. Ç., Öğüt, N. U., Mutlu, B., Çeçen, E., and Can, E.	Revista da Associacao Medica Brasileira	2021	Turkey	Translation and adaptation	41	COVERS (Test), PIPP and NIPS (Control)	Preterm and term infants	Validation of COVERS, in Turkey	NICU
